# Molecular regulation of pancreatic stellate cell function

**DOI:** 10.1186/1476-4598-3-26

**Published:** 2004-10-06

**Authors:** Robert Jaster

**Affiliations:** 1 Department of Medicine, Division of Gastroenterology, Medical Faculty, University of Rostock, E.-Heydemann-Str. 6, 18057 Rostock, Germany

## Abstract

Until now, no specific therapies are available to inhibit pancreatic fibrosis, a constant pathological feature of chronic pancreatitis and pancreatic cancer. One major reason is the incomplete knowledge of the molecular principles underlying fibrogenesis in the pancreas. In the past few years, evidence has been accumulated that activated pancreatic stellate cells (PSCs) are the predominant source of extracellular matrix (ECM) proteins in the diseased organ. PSCs are vitamin A-storing, fibroblast-like cells with close morphological and biochemical similarities to hepatic stellate cells (also known as Ito-cells). In response to profibrogenic mediators such as various cytokines, PSCs undergo an activation process that involves proliferation, exhibition of a myofibroblastic phenotype and enhanced production of ECM proteins. The intracellular mediators of activation signals, and their antagonists, are only partially known so far. Recent data suggest an important role of enzymes of the mitogen-activated protein kinase family in PSC activation. On the other hand, ligands of the nuclear receptor PPARγ (peroxisome proliferator-activated receptor γ) stimulate maintenance of a quiescent PSC phenotype. In the future, targeting regulators of the PSC activation process might become a promising approach for the treatment of pancreatic fibrosis.

## Review

Excessive production of connective tissue molecules forming the extracellular matrix (ECM) is a pathological process relevant to diseases of many organ systems, including liver, lung, kidney, bowel and pancreas. The resulting fibrosis frequently leads to a progressive loss of specific organ functions. In the past two decades, fibrogenesis has been intensively studied by a large number of laboratories, and a great deal of scientific information has been accumulated regarding the pathogenesis of fibrosis in various organs. Until a few years ago, pancreatic fibrosis, however, remained an exception: although known for a long time as a central pathological feature of both chronic pancreatitis and pancreatic cancer [1,2], its cellular and molecular basics remained obscure. This situation has changed significantly since the identification of a fibroblast-like cell type in the pancreas with close similarities to hepatic stellate cells (HSCs; also called Ito cells) [3,4], the predominant source of ECM in the fibrotic liver [5,6]. In the meantime, it has become increasingly clear that these stellate cells of the pancreas (named pancreatic stellate cells; PSCs) are the principle effector cells in pancreatic fibrosis. In the following sections, I will focus on (I) the current understanding of the role of PSCs in fibrogenesis, (II) extracellular signals involved in PSC activation, (III) intracellular mediators of activation signals in PSCs, (IV) future directions of research, and (V) activated PSCs as a target for antifibrotic therapies.

### Pancreatic stellate cells and their role in pancreatic fibrogenesis

Both chronic pancreatitis and pancreatic cancer are accompanied by an organ fibrosis [1,2]. The progressive replacement of pancreas-specific tissue by ECM-rich connective tissue leads to the development of an exocrine and endocrine insufficiency of the gland. So far, specific therapies to prevent, retard or even reverse this process are not available.

Fibroblast activation has been reported to be a common event in pancreatitis already more than a decade ago [7-9], but the basic matrix producing cell type in the pancreas remained to be identified. In 1997, Saotome et al. [10] described the isolation of periacinar fibroblast-like cells from human pancreas. The cells displayed some characteristics of activated myofibroblasts, e.g. expression of α-smooth muscle actin (α-SMA) and synthesis of ECM proteins. One year later, Bachem et al. [3] and Apte et al. [4] found that vitamin A-storing cells resembling hepatic stellate cells can be isolated from human and rat pancreas. In the healthy organ, PSCs comprise about 4% of all pancreatic cells and show a periacinar distribution. They can be identified by the presence of retinoid-containing cytoplasmic lipid droplets and by immunostaining for cytoskeletal proteins such as desmin and glial fibrillary acidic protein [4]. In culture, pancreatic stellate cells readily grow [4] and change from a quiescent phenotype to a myofibroblast-like cell expressing α-SMA and producing large amounts of the ECM proteins collagen type I and III, fibronectin as well as laminin [3]. This activation process is accompanied by a loss of the characteristic retinoid-containing fat droplets [3,4]. Together, these *in vitro *data gave rise to the hypothesis that PSCs might play a pivotal role in pancreatic fibrogenesis.

In the meantime, this hypothesis has been supported by the results of several *in vivo *studies using experimental models of pancreatic fibrosis: Infusion of trinitrobenzene sulfonic acid (TNBS) into the pancreatic duct of rats causes a pancreatic necroinflammation followed by fibrosis [11]. In TNBS-treated rats, areas of pancreatic fibrosis colocalized with α-SMA-positive cells, suggesting the presence of activated PSCs. Furthermore, dual staining techniques indicated that these α-SMA-positive cells were the main source of collagen in the fibrotic pancreas [12]. Importantly, very similar data were obtained when pancreatic tissue from patients with chronic pancreatitis was analyzed [12]. Another well-established model of fibrogenesis involves the administration of a single intravenous dose of dibutyltin dichloride (8 mg/kg body weight), resulting in the development of a chronic pancreatitis associated with fibrosis [13]. Time course studies of DBTC-induced chronic pancreatitis revealed an early activation of PSCs that preceded development of fibrosis [14]. In mice, repeated intraperitoneal application of supraphysiological cerulein doses causes a pancreatic injury and, subsequently, fibrosis [15,16]. In agreement with the data mentioned above, collagen gene expression was colocalized to PSCs [16]. Overexpression of transforming growth factor-beta (TGF-β) 1 in transgenic mice has been shown to be associated with increasing deposition of ECM in the pancreas. In parallel with the development of fibrosis, the number of PSCs in the pancreas increased [17].

Recently, it has also been suggested that PSCs contribute to regeneration early after acute necrotising pancreatitis in humans [18].

Together, *in vitro *and *in vivo *data suggest that PSCs are essentially involved in the development of pancreatic fibrosis.

### Extracellular signals involved in pancreatic stellate cell activation

Based on the results of various recent studies, extracellular factors involved in PSC activation may be divided into two major groups: (I) cytokines/growth factors [3,19-22] and (II) ethanol and its metabolites, most of all acetaldehyde [23].

Cytokines stimulating PSC activation include platelet-derived growth factor (PDGF) [3,19,21,22], the TGF-β family members TGF-β1 [3,19,21,22] and activin A (24), TGF-alpha [3,22], basic fibroblast growth factor [3,22], tumor necrosis factor-α (TNF-α) [22], interleukin (IL)-1 [20] and IL-6 [20]. While TGF-β1 efficiently promotes ECM synthesis [3,19,21,22], PDGF is considered to be the most effective mitogen [22]. Furthermore, PDGF also enhances the migratory capacity of PSCs [25]. Potential sources of cytokines stimulating PSC activation in the inflamed pancreas are, for example, activated macrophages (secreting TGF-β1) [26], platelets (containing PDGF and TGF-β1) [21], and possibly acinar cells (expressing, among other cytokines, TNF-α [27], IL-1 and IL-6 [28]). Importantly, PSCs themselves are capable of synthesizing cytokines such as TGF-β1 [29,30], activin A [24] and IL-1 [31]. These observations suggest the existence of autocrine loops that may contribute to the perpetuation of PSC activation after an initial exogenous signal, thereby promoting the development of fibrosis.

Recent studies have also implicated the pancreatic renin-angiotensin system [32,33] in pancreatic fibrogenesis. Thus, application of the angiotensin-converting enzyme inhibitor lisinopril [34], as well as the angiotensin II receptor antagonist candesartan [35], suppressed pancreatic inflammation and fibrosis in an animal model of spontaneously occurring chronic pancreatitis, Wistar Bonn/Kobori rats. In angiotensin II receptor type 1a-deficient (AT1a(-/-)) mice, pancreatic fibrosis induced by repeated episodes of acute pancreatitis (following cerulein injections) was found to be attenuated [36]. *In vitro*, angiotensin II (ATII) stimulates PSC proliferation [37,38] and induces cell contraction [38].

Cytokines that act as antagonists of PSC activation have not been systematically studied so far. Recently, it has been shown that IFN-α protects *hepatic *stellate cells from lipid peroxidation by enhancing biological activities against oxidative stress, resulting in an inhibition of activation [39]. Furthermore, antiproliferative effects of IFN-α [40], IFN-β and IFN-γ [41] on HSCs have been reported. On the other hand, IFN-α also inhibits spontaneous apoptosis of activated HSCs [40]. The effects of interferons on pancreatic fibrogenesis remain to be characterized.

Although it is known for a long time that chronic pancreatitis, associated with fibrosis, is a serious complication of alcohol abuse, the pathogenesis of alcoholic pancreatitis still remains to be fully elucidated [reviewed in [42]]. In recent studies, the question has been addressed how long-term alcohol consumption is linked to PSC activation and fibrosis. It has been proposed that the profibrogenic effects of ethanol are in part mediated by PSC-activating proinflammatory cytokines released during episodes of alcoholic pancreatitis (associated with necroinflammation) [43]. Furthermore, *in vitro *data suggest that ethanol directly acts on PSCs and induces activation [23]: Cultured PSCs respond to ethanol application by increased α-SMA expression and collagen synthesis. Stimulatory effects of ethanol were detectable both in already activated and still quiescent PSCs. The cells express alcohol dehydrogenase, indicating that they are capable of ethanol oxidation and generation of its metabolite acetaldehyde. Very likely, induction of oxidant stress in PSCs contributes to the profibrogenic effects of ethanol [23]. Although the exact chain of events linking ethanol abuse to pancreatic inflammation and PSC activation remains to be described, it is likely that both direct and indirect (cytokine-mediated) effects of ethanol on PSCs are involved in the development of pancreatic fibrosis.

### Intracellular transduction of activation signals

In the past two years, analysis of signal transduction pathways regulating PSC function has become a focus of attention. As detailed below, identification of signaling molecules that play a crucial role in PSC activation is a promising approach for the development of therapeutic strategies to inhibit pancreatic fibrosis. It is therefore envisaged that the *systematic *elucidation of signaling pathways in PSCs will also be one of the most important issues for future research. So far, research regarding intracellular signaling in PSCs has focused on two main aspects: the role of enzymes of the mitogen-activated protein kinase (MAPK) family and the transcriptional control of PSC activation.

MAPKs are a family of serine/threonine specific protein kinases with a wide range of biological functions in the regulation of fundamental cellular processes, including gene expression, proliferation and cell survival/apoptotic cell death [44-46]. In mammalian cells, three major MAPK families (extracellular signal-regulated kinases [ERKs], c-Jun N-terminal kinase [JNK] and p38) have been identified [45], and all of them have recently been studied with respect to the regulation of PSC activation. The best-characterized ERKs, ERK 1 and 2, are activated through a well-established pathway (induced by many growth factors) that involves, among several other cytosolic proteins, the small G-protein Ras and the serine/threonine-specific protein kinase Raf-1 [45]. In the process of PSC activation induced by sustained culture, ERK 1/2 activation is an early event that precedes exhibition of a myofibroblastic phenotype [47]. The strong PSC mitogen PDGF induces an activation of ERK 1/2, and inhibition of signaling through the Ras-Raf-ERK signaling cascade attenuates PSC proliferation [47-49]. It has also been shown that exposure of PSCs to ethanol and acetaldehyde is accompanied by a fast [50] and long-lasting [51] ERK 1/2 activation.

The other two major MAP kinase pathways, involving JNK and p38, are well-established mediators of signals induced by pro-inflammatory cytokines and cellular stressors (e.g., oxidant stress, UV irradiation) [52]. In PSCs, both JNK and p38 are activated in response to ethanol/acetaldehyde exposure [50,51]. Inhibition of p38 enzymatic activity interferes with ethanol-induced myofibroblastic transdifferentiation of PSCs [51]. The p38 signaling pathway has also been implicated in the mediation of the mitogenic PDGF effect and in the induction of PSC activation induced by sustained culture [53]. Incubation of freshly isolated PSCs with the JNK inhibitor SP600125 attenuates proliferation of the cultured cells [54]. MAP kinase pathways have also been shown to be involved in ATII signaling in PSCs [37,38]. Together, these data support the hypothesis that MAPKs are key mediators of activation signals in PSCs.

Two other intracellular signal transduction pathways that have recently been studied regarding their role in PSC activation are the phosphatidylinositol 3 (PI 3)-kinase and the Rho-Rho kinase (ROCK) pathway. The results suggest that PI 3-kinase activity is required for PDGF-stimulated PSC migration but not proliferation [49,55]. The Rho-ROCK pathways was shown to be involved in the activation process of PSCs *in vitro *by regulating the actin cytoskeleton [56].

Cytokine and growth factor receptors exert their effects on the expression of target genes through signaling cascades that regulate the activity of a characteristic set of transcription factors. Recently, the group of the author has analyzed the activation profiles of activator protein (AP)-1 [57,58], signal transducer and activator of transcription (STAT) 3 [59] and nuclear factor (NF)-κB [60,61] in the course of PSC activation induced by sustained culture. AP-1 and NF-κB displayed an earlier maximum of DNA binding activity than STAT3 [62]. Further experiments revealed that phenotypic transition of PSCs towards myofibroblasts was accompanied by characteristic changes of AP-1 complex composition (increase of the JunD content relative to the one of JunB) [62]. DNA binding of AP-1 in PSCs is induced by PDGF, suggesting AP-1 activation as an important step in the process of PSC activation [47].

In the transduction of TGF-β receptor-derived signals into the nucleus, Smad transcription factors play a central role [63,64]. Studies by Ohnishi and co-workers revealed that TGF-β1 stimulated PSC activation (indicated by increased α-SMA expression) in a Smad2-dependent manner, while Smad3 was required for TGF-β1-induced growth inhibition [65]. Interestingly, exogenous TGF-β1 increased TGF-β1 mRNA expression in PSCs through an ERK-dependent but Smad2/3-independent pathway. Together, these data suggest distinct roles of Smad2-, Smad3- and ERK-dependent pathways in TGF-β1 regulation of PSC functions. Based on recently published data on HSC biology [66], it can be hypothesized that Smad7, a negative regulator of TGF-β signaling, might act as a transcriptional inhibitor of PSC activation, but so far experimental evidence has not been presented.

Recent studies have implicated the nuclear hormone receptor peroxisome proliferator-activated receptor γ (PPARγ) in the inhibition of stellate cell activation in liver [67-69] and pancreas [69]: The PPARγ ligands 15-deoxy-Δ12,14-prostaglandin J_2 _and troglitazone (an antidiabetic drug of the thiazolidinedione group) act as antagonists of PSC activation *in vitro *that decrease cell proliferation and expression of α-SMA [70]. In Wistar Bonn/Kobori rats, troglitazone attenuates pancreatic inflammation and fibrosis [71]. The antifibrotic effect of the drug, however, was found to be in part mediated via a PPARγ-independent mechanism [72]. Thus, the precise role of PPARγ in pancreatic fibrogenesis remains to be elucidated in further studies.

### Open questions with respect to PSC biology and pathology

While the role of activated PSCs in pancreatic fibrosis is well established, the physiological functions of their quiescent precursors are less well understood. Importantly, PSCs are not only a source of ECM but also of matrix-degrading enzymes of the MMP (matrix metalloproteinases) family and their inhibitors (tissue inhibitors of matrix metalloproteinases, TIMPs). Thus, PSCs have been shown to secrete MMP-2, MMP-9 and MMP-13 and to express TIMP-1 and TIMP-2 [73]. It appears therefore likely that PSCs participate in the regulation of matrix turnover in the healthy pancreas.

The embryonic origin of PSCs still remains to be determined. Very recently, Seaberg et al. [74] reported the clonal identification of multipotent precursors from adult mouse pancreas that generate neural and pancreatic lineages, including β-like cells and pancreatic stellate cells. With regard to PSC biology, one implication of this pioneer study is that PSCs share with exocrine and endocrine pancreatic lineages a common progenitor cell. Kruse et al. [75] have described the isolation and culture of undifferentiated pancreatic cells, capable of extended self-renewal and spontaneous differentiation into cells of all three germ layers. The relationships between these cells, which were described as stellate-like cells, and PSCs are currently unknown and should be further studied using clonal cell populations.

Until now, the physiological consequences of vitamin A-storage in PSCs remain unclear. It has recently been shown by the group of the author that the vitamin A derivate all-trans retinoic acid has complex effects on PSC function and acts, at least in part, as an antagonist of the activation process [76]. It is, therefore, tempting to speculate that retinoic acids, through the binding to their nuclear receptors and the regulation of gene expression, are involved in the maintenance of a quiescent PSC phenotype. In this scenario, the loss of retinoids in the course of PSC activation might be not an epiphenomenon but an essential prerequisite.

In the past, research regarding PSC biology has almost exclusively focused on the molecular basics of the activation process. However, given that participation in regeneration after pancreatic injury is an important function of activated PSCs, it is apparent that a disturbance of the inactivation or elimination of activated PSCs, rather than PSC activation itself, is the pathological process that leads to fibrosis. So far, it has not been systematically studied whether activated PSCs are capable of returning into a quiescent stage after fulfilling a repair function. Alternatively, elimination by apoptosis might be important in terminating the wound-healing response after pancreatic injury [77].

Finally, work on the complex relationships between PSCs and pancreatic tumor cells is still in its infancy. Very likely, activation of PSCs not simply accompanies tumor progression but plays an active role in this process. Thus, it has been recently been shown that pancreatic cancer growth and progression is accelerated through complex functional interactions between carcinoma cells and PSCs [78]. Furthermore, the increased deposition of connective tissue in pancreatic carcinoma was suggested to be the result of a paracrine stimulation of PSCs by cancer cells [79]. Interestingly, TGF-β1-transfected pancreatic tumor cells have been demonstrated to induce a rich stroma after orthotopical transplantation in the nude mouse pancreas [80]. Considering the established role of TGF-β1 in PSC activation [3,19,21,22], it appears likely that the cytokine is a key effector in tumor-associated pancreatic fibrosis. It is easy to predict that the further analysis of PSC activation in pancreatic cancer will be an important research area in the future.

Studies on PSC biology are still hampered by the limited availibility of primary cells. Possibly, recently established pancreatic stellate cell lines [81,82] will be helpful in overcoming this problem.

### Therapeutic implications

Given that activated PSCs are the principle effector cells in pancreatic fibrosis, targeting PSCs might become a promising therapeutic approach. Principle strategies that can be envisaged include an interruption/reversion of the activation process as well as an elimination of activated PSCs, e.g. through an induction of apoptosis. So far, potential antifibrotic drugs have been mainly tested in models of liver fibrosis (reviewed in [83]). The existence of common mechanisms in the development of liver and pancreatic fibrosis (particularly, the key role of activated stellate cells), however, suggests that at least some of these drugs may also be effective inhibitors of fibrogenesis in the pancreas. In this regard, the efficiency of substances interfering with the action of stellate cell mitogens (e.g., PDFG), or cytokines stimulating ECM synthesis (especially TGF-β), should be tested in animal models of pancreatic fibrosis. The inhibitory effects of an angiotensin-converting enzyme inhibitor [34], as well as an ATII receptor antagonists [35], on pancreatic fibrosis need to be further evaluated. Interesting candidates are also cytokines that display inhibitory effects on *hepatic *stellate cell activation, such as interferons.

As described above, studies on the regulation of PSC activation at the intracellular level have identified key mediators of stimulatory and inhibitory signals. Targeting molecules such as PPARγ, MAP kinases, PI 3-kinase, or Smad proteins might become an important approach for the treatment of pancreatic fibrosis in the future. Further progress in the development of antifibrotic therapies can be expected from the ongoing elucidation of the molecular principles of PSC activation.

## Conclusions

**Figure 1:**
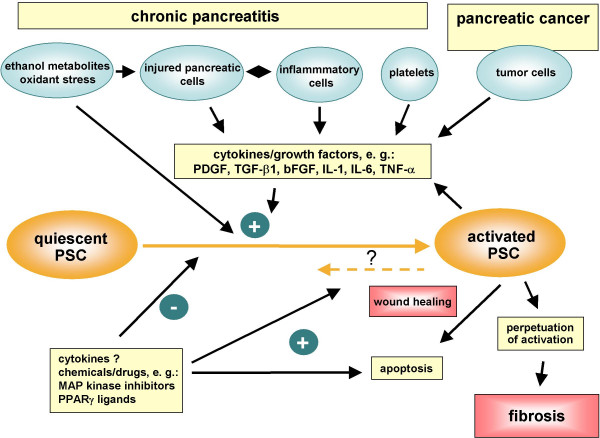
Pancreatic stellate cell activation in chronic pancreatitis and pancreatic cancer. Pancreatic stellate cells are activated by profibrogenic mediators, such as ethanol metabolites and cytokines/growth factors. Perpetuation of stellate cell activation under persisting pathological conditions results in pancreatic fibrosis.

PSCs play a crucial role in pancreatic fibrogenesis (Figure 1). Ethanol metabolites and cytokines such as PDGF and TGF-β are key activators of PSCs. The intracellular regulation of PSC activation is incompletely characterized. MAP kinase signaling cascades are involved in the transduction of activation signals, while PPARγ ligands induce a quiescent PSC phenotype. The recent progress in the understanding of the cellular and molecular basics of pancreatic fibrosis will facilitate the development of therapeutic strategies to inhibit pancreatic fibrosis.
